# International Variability in the Diagnosis and Management of Ankyloglossia Among Dental Professionals: A Cross-Sectional Study

**DOI:** 10.3390/dj14070436

**Published:** 2026-07-14

**Authors:** Elvira Ferrés-Amat, Carlos Perez-Torres, Maria Carmela Giovannelli, Ana Vallés-Creixell, Constanza Sanchez-Davila, Paulina Aliaga Sancho, Claudia Naranjo Camilla, Ana Luísa Costa, Ana Veloso Duran, Lluis Giner-Tarrida, Francisco Guinot

**Affiliations:** 1 Paediatric Dentistry Department, Faculty of Dentistry, Universitat Internacional de Catalunya, 08195 Barcelona, Spain; eferresamat@uic.es (E.F.-A.); fguinot@uic.es (F.G.); 2 Escuelas de Odontologia, Universidad San Francisco de Quito, Quito 170901, Ecuadorpaliaga@usfq.edu.ec (P.A.S.); 3 Faculty of Dentistry, Universidad de los Andes, Santiago 12455, Chile; cnaranjo@uandes.cl; 4 Paediatric and Preventive Dentistry Institute, Faculty of Medicine, University of Coimbra, 3004-531 Coimbra, Portugal; 5 Center for Innovation and Research in Oral Sciences, Faculty of Medicine, University of Coimbra, 3004-531 Coimbra, Portugal; 6 Dentistry Department, Faculty of Dentistry, Universitat Internacional de Catalunya, 08195 Barcelona, Spain; lginer@uic.es; 7 Service of Pediatric Dentistry, Hospital HM Nens, HM Hospitales, 08009 Barcelona, Spain; 8 HM Hospitals Health Research Institute, 28015 Madrid, Spain

**Keywords:** ankyloglossia, tongue tie, lingual frenulum, infant

## Abstract

**Background/Objectives**: Ankyloglossia diagnosis and management remain widely debated in dentistry, with variability reported in clinical decision-making and treatment approaches. Limited international evidence exists comparing how dental professionals diagnose and manage this condition in early childhood. This study aimed to compare the knowledge, diagnostic criteria, and clinical management of ankyloglossia among dental professionals in six countries and to assess whether shared diagnostic and management approaches exist for babies (≤6 months) and infants (>6 months). **Methods**: A multicountry cross-sectional study was conducted using a validated 27-item online questionnaire distributed between February 2023 and February 2025 to dental professionals in Spain, Italy, Portugal, Brazil, Ecuador, and Chile. Participants included paediatric dentists, oral surgeons, general dentists, and other dental specialists. Only fully completed questionnaires were analyzed. Primary outcomes included self-reported diagnostic practices, use of classification systems, and management strategies for ankyloglossia. Secondary outcomes included professional training, interdisciplinary collaboration, surgical techniques, and pain management practices. **Results:** A total of 1188 dental professionals participated. Overall, 80.7% reported routinely examining the lingual frenulum, while 42.4% used formal classification systems. Tongue mobility assessment was the most commonly reported diagnostic method, with significant variation across countries and professional groups (*p* < 0.001). Specific training in diagnosis was reported by 67.2% of participants, whereas 50.6% reported treatment training, with marked inter-country differences (*p* < 0.001). Surgical intervention was the most commonly reported management strategy (38.7%), particularly in babies ≤ 6 months, with frenotomy/frenulotomy being the predominant procedure. Considerable variability was observed in surgical techniques, pain management, and recommendations for adjunctive therapies. **Conclusions**: Substantial international variability exists in the diagnosis, classification, and management of ankyloglossia among dental professionals. These findings highlight gaps in training and the absence of standardized, evidence-based clinical guidelines, underscoring the need for consensus-driven protocols to support consistent and multidisciplinary clinical care.

## 1. Introduction

The tongue is a dynamic organ essential for multiple oral and systemic functions, including sucking, chewing, swallowing, speech, breathing, taste, and craniofacial development. Under normal physiological conditions, the tongue rests against the hard palate behind the maxillary anterior teeth, contributing to balanced orofacial growth and functional harmony [1]. The lingual frenulum is a submucosal band of connective tissue located beneath the tongue, inserting near its tip or undersurface. It is considered a vestigial embryological structure that contributes to maintaining equilibrium between oral tissues during growth and development [1,2,3].

Ankyloglossia, commonly known as tongue-tie, is defined by both anatomical and functional criteria; however, a universally accepted definition remains lacking. It is generally described as a short, thickened, or abnormally positioned lingual frenulum that restricts tongue mobility [2,4]. The International Association of Tongue-Tie Professionals describes ankyloglossia as a persistent embryologic tissue remnant resulting from incomplete apoptosis, producing a shortened or anteriorly positioned frenulum that limits tongue movement [3,5]. Reported prevalence in the general population ranges from 4% to 10.7%, with increasing detection rates likely associated with broader diagnostic definitions and the absence of standardized assessment criteria [6]. Among newborns, prevalence is estimated at approximately 5%, although considerable variation exists depending on study methodology [7]. No consistent ethnic predilection has been established, but males appear to be affected more frequently than females, with an approximate ratio of 3:1 [8]. Genetic influences have been suggested, including possible X-linked inheritance patterns and associations with syndromes such as Simpson–Golabi–Behmel, Opitz, Orofacial Digital, Beckwith–Wiedemann, and X-linked cleft palate syndromes [7,8,9].

Diagnosis of ankyloglossia remains challenging due to the lack of standardized screening protocols and classification systems. Accurate clinical evaluation requires a comprehensive understanding of normal tongue anatomy and function, as alterations in frenulum morphology may impair mobility and oral function [7,10]. Several diagnostic tools and classification protocols have been proposed, including those developed by Kotlow, Coryllos, Hazelbaker, Lalakea, Horton, Ruffoli, the Bristol Tongue Assessment Tool, and the Lingual Frenulum Protocol for Infants; however, their reliability and universal applicability remain subjects of ongoing debate [1,2,3,4,5,6,7,8,9,10,11]. The variability among these systems contributes to inconsistent diagnosis and may influence clinical decision-making.

Management of ankyloglossia is similarly controversial, particularly regarding indications for treatment and the choice between conservative and surgical approaches. While some clinicians advocate early surgical intervention to prevent feeding or speech difficulties, others recommend conservative monitoring unless functional impairment is clearly demonstrated. Recent recommendations from the American Academy of Pediatric Dentistry emphasize the importance of individualized, function-based management and interdisciplinary collaboration [11]. At present, no single surgical technique has been established as the definitive standard of care, largely due to limited high-quality evidence supporting one method over another. Common surgical procedures include frenotomy, involving a simple incision of the frenulum; frenectomy or frenulectomy, involving complete excision of the frenulum; and frenuloplasty, which includes excision and repositioning of surrounding tissues to improve mobility [11,12,13].

Despite increasing clinical awareness of ankyloglossia, substantial variability persists in diagnostic practices, classification methods, and treatment strategies among dental professionals. The absence of standardized diagnostic criteria and evidence-based treatment guidelines contributes to inconsistent clinical decision-making and potentially variable patient outcomes. Differences in professional education, training, and clinical experience may further contribute to regional and professional disparities in management approaches. However, large-scale international comparisons of professional knowledge and clinical practices remain limited.

Therefore, the aim of this study was to collect, analyze, and compare the knowledge, diagnostic criteria, and clinical management practices of dental professionals in Spain, Italy, Portugal, Brazil, Ecuador, and Chile regarding ankyloglossia, and to determine whether shared diagnostic and management approaches exist for babies (≤6 months) and infants (>6 months). The findings of this study highlight substantial international variability in clinical practice and underscore the need for standardized, evidence-based guidelines to support consistent and multidisciplinary patient care.

## 2. Materials and Methods

### 2.1. Study Design and Population

This multicountry cross-sectional study was conducted between February 2023 and February 2025 using an online questionnaire consisting of 27 items designed to assess and compare the knowledge, diagnostic criteria, and treatment preferences related to ankyloglossia among dental professionals in Spain, Italy, Ecuador, Portugal, Chile, and Brazil. The study population consisted of licensed dental professionals, including pediatric dentists, oral surgeons, general dentists, and other dental specialists. Pediatric dentists and oral surgeons were analyzed separately due to their primary role in diagnosing and treating ankyloglossia. Sample size calculation was performed using an estimated proportion of *p* = *q* = 0.5 and a margin of error of 7%, assuming no sample loss. Based on these parameters, a minimum sample size of 166 participants per country was required to ensure adequate statistical power and precision for comparisons between countries. The inclusion criteria were dental professionals practicing in the participating countries and completing the questionnaire within the designated timeframe. The exclusion criteria included healthcare professionals from non-dental fields, undergraduate students, late submissions, and incomplete surveys.

### 2.2. Questionnaire Development and Validation

The electronic questionnaire was developed by the Department of Pediatric Dentistry at the Universitat Internacional de Catalunya (UIC), Barcelona, Spain, using standardized terminology from the American Academy of Pediatric Dentistry (AAPD) [11].

The questionnaire consisted of four sections:

(1) Demographic and professional background;
(2) Knowledge of ankyloglossia, including etiology, clinical features, and consequences;
(3) Cinical diagnostic approaches, including examination techniques and classification criteria;
(4) Treatment practices and referral preferences for neonates and infants.

To ensure linguistic and cultural equivalence across participating countries, the original Spanish version was translated into English, Portuguese, and Italian and subsequently back-translated by native speakers affiliated with each participating institution. Validation followed the methodology described by Maneesriwongul et al. [12], ensuring semantic, conceptual, content, and technical equivalence across languages.

A pilot study involving 15–20 dental professionals per country was conducted to assess clarity, reliability, and cultural relevance. Concordance and kappa indices were calculated to evaluate interobserver agreement, and minor wording adjustments were implemented to improve clarity and consistency.

### 2.3. Survey Distribution and Data Collection

The questionnaire was distributed electronically through professional networks, academic mailing lists, and social media platforms targeting dental professionals in the participating countries. Participation was voluntary, and all responses were collected anonymously to ensure confidentiality. Only fully completed questionnaires submitted within the study period were included in the final analysis.

### 2.4. Ethical Considerations

The study protocol was approved by the Research Ethics Committee of the Universitat Internacional de Catalunya (UIC), Barcelona, Spain (approval code: ODP-INVI-2024-02), as well as by the corresponding ethics committees in each participating country.

All participants received detailed information regarding the purpose of the study and provided informed consent prior to participation. Data were collected and processed anonymously in accordance with applicable data protection regulations, including Organic Law 3/2018 on the Protection of Personal Data and Guarantee of Digital Rights.

### 2.5. Statistical Analysis

Descriptive statistics (means, frequencies, and percentages) summarized demographic and response data using Jamovi statistical software (Version 2.3.21; The Jamovi Project, Sydney, NSW, Australia). Chi-square and Fisher’s exact tests were applied to compare proportions across countries and professional categories. Statistical significance was determined at *p* < 0.001. Subgroup analyses explored differences according to specialization and country to evaluate how cultural and healthcare system factors influenced knowledge and clinical management of ankyloglossia.

## 3. Results

A total of 1.188 answered surveys were obtained from professionals across six countries: Spain 222, Italy 220, Portugal 179, Brazil 175, Ecuador 220, and Chile 174. Most respondents were women (69.4%) with a mean age of 40.2 years. Pediatric dentists represented the largest group (39.6%), followed by general dentists (27.9%), oral surgeons (13.7%), and other specialists (18.9%).

Specific training in diagnosing ankyloglossia was reported by 67.2%, mainly through postgraduate education (28.7%). Only 50.6% received treatment training, with large country-level differences: Brazil (73.1%) and Spain (54.1%) reported the highest rates, while Portugal had the lowest (30.7%) (*p* < 0.001).

Regarding clinical assessment, 80.7% reported routinely examining the lingual frenulum, most often using a combined method of observation and palpation (69.4%), followed by observation alone (22.8%). A formal classification system was used by 42.4%, while 57.6% used none. Methods varied significantly by country and specialty (*p* < 0.001). Pediatric dentists overwhelmingly favored the combined approach (>80% in all countries), while oral surgeons also reported high use (Brazil 92.0%, Italy 88.9%, Chile 86.8%). General dentists were more variable: in Portugal, only 38.3% used both techniques and 34.6% did not examine the frenulum; Spain showed a balanced split between visual-only (38.3%) and combined (41.7%). Almost half of respondents (47.7%) reported not using a formal classification system. Among users, tongue mobility assessment was most common (19.4%), followed by the Bristol Tongue Assessment Tool (9.8%, mainly pediatric dentists), the Martinelli “Teste da Linguinha” (9.0%, frequent in Brazil, Portugal, Chile), Coryllos (5.4%), and HATLFF (4.5%). Patterns varied by country and specialty, reflecting the absence of standardized diagnostic practice (*p* < 0.001) (Table 1).

**Table 1:** Ankyloglossia Classification tool per country.

Classifications		BRAZIL	CHILE	ECUADOR	ITALY	PORTUGAL	SPAIN	TOTAL
Bristol (BTAT)	% within column	42.30%	05.70%	05.50%	02.30%	03.40%	04.50%	09.80%
Coryllos	% within column	02.30%	06.90%	03.60%	04.10%	00.60%	13.50%	05.40%
Evaluation based on tongue mobility	% within column	08.60%	15.50%	25.00%	28.40%	14.50%	20.7%	19.40%
Hazelbaker (HATLFF)	% within column	04.00%	05.70%	02.70%	04.10%	02.20%	07.70%	04.50%
I do not use any classification	% within column	20.60%	56.30%	50.90%	45.90%	66.50%	45.90%	47.70%
Other	% within column	00.00%	01.10%	05.50%	09.60%	01.70%	05.00%	04.10%
Taste da linguinha (Martinelli)	% within column	22.30%	08.60%	06.80%	05.50%	12.20%	02.70%	09.00%
Total	% within column	100.00%	100.00%	100.00%	100.00%	100.00%	100.00%	100.00%

X^2^ Tests



Valuedf*p*X^2^42230<0.001N1188



Collaboration with other healthcare professionals also varied: 49.7% reported regular collaboration, 30.7% occasional, and 19.6% none (*p* < 0.001). Rates were highest in Chile (60.3%), Brazil (58.3%), and Spain (57.7%), but lowest in Portugal (25.1%), where 40.8% reported no collaboration. Pediatric dentists showed the highest collaboration (66.6%), followed by oral surgeons (57.7%), while general dentists (34.1%) and “Other” specialists (27.2%) most often reported none.

Breastfeeding assessment in infants under six months was widely reported (86.4%), though again variable (*p* < 0.001). Rates were highest in Brazil (94.3%), Italy (91.7%), and Chile (91.4%), and lowest in Portugal (69.3%), where nearly one-third did not include this step.

Surgical intervention was the most frequent management strategy (38.7%), followed by referral (36.0%), monitoring (14.6%), and no treatment (7.8%) (*p* < 0.001). Brazil reported the highest surgical rate (69.1%), while Portugal had the lowest (23.5%) and the highest non-treatment (40.2%). Pediatric dentists (56.2%) and oral surgeons (54%) were most likely to perform surgery, while general dentists more often referred or opted not to treat.

In infants up to six months, frenotomy/frenulotomy was the most common surgical procedure (59.3%), while 30.2% reported no intervention (*p* < 0.001). Brazil (83.4%) and Italy (77.1%) had the highest frenotomy rates, whereas Portugal showed the highest non-performance (78.2%). Pediatric dentists (73.6%) and oral surgeons (64.4%) most frequently performed frenotomy, while general dentists (41.4%) and “Other” professionals (54.9%) most often reported no intervention.

Scissors were the preferred surgical instrument (43.1%), particularly in Brazil (80.6%) and Ecuador (55.9%), whereas laser (17.5%) and electrosurgery (11.2%) were less commonly used. Pain management practices also varied: 32.3% reported no pain control, while others used combined topical and infiltrative anesthesia (21.4%), topical alone (14.4%), infiltration alone (6.5%), or breastfeeding (15.7%). Pediatric dentists most frequently employed pain control methods, oral surgeons reported the highest use of general anesthesia (12.9%), and general dentists along with “Other” professionals most often reported no use of anesthesia or analgesia (Table 2).

**Table 2:** Knowledge and approach of ankyloglossia in babies (≤6 months).

Surgical Techniques	SPAIN	PORTUGAL	ITALY	BRAZIL	ECUADOR	CHILE
Frenotomy	56.70%	19.60%	77.10%	83.40%	66.80%	47.70%
Frenectomy	08.60%	01.70%	06.40%	14.30%	06.40%	05.70%
Frenuloplasty	01.40%	0.60%	1.80%	02.30%	10.00%	02.90%
Not perform	33.30%	78.10%	14.70%	00.00%	16.80%	43.70%
Total	100.00%	100.00%	100.00%	100.00%	100.00%	100.00%
Surgical Modalities						
Conventional	41.90%	10.10%	38.50%	80.60%	55.90%	30.50%
Laser	11.30%	11.70%	44.50%	08.00%	11.80%	14.30%
Electrosurgery	12.10%	01.10%	17.00%	07.40%	16.80%	09.80%
Not perform	34.70%	77.10%	00.00%	04.00%	15.50%	45.40%
Total	100.00%	100.00%	100.00%	100.00%	100.00%	100.00%
Pain control						
General Anesthesia	00.50%	00.00%	01.80%	00.00%	05.90%	09.80%
Glucose saline solution	03.20%	00.00%	06.40%	00.00%	00.50%	00.00%
Immediate Breastfeeding	25.20%	02.80%	18.80%	13.10%	16.40%	14.90%
Local cold stimulant	00.90%	00.60%	02.80%	00.00%	00.90%	00.60%
Infiltrative local anesthesia	05.90%	05.60%	03.70%	08.60%	07.70%	08.00%
Infiltrative local anesthesia and sedation	01.80%	00.60%	06.40%	02.90%	06.80%	04.00%
Topical local anesthesia	11.30%	02.80%	27.50%	23.40%	13.20%	06.30%
Topical local anesthesia and infiltrative local anesthesia	13.50%	07.80%	21.10%	45.70%	30.00%	10.30%
Not use	37.70%	79.80%	11.50%	06.30%	18.60%	46.10%
Total	100.00%	100.00%	100.00%	100.00%	100.00%	100.00%

**Surgical Techniques****Surgical Modalities****Pain Control**X^2^ Tests


X^2^ Tests


X^2^ Tests



Valuedf*p*
Valuedf*p*
Valuedf*p*X^2^37415<0.001X^2^54715<0.001X^2^52040<0.001N1188

N1188

N1188



In infants over six months, frenotomy/frenulotomy (31.6%) remained the most frequently reported procedure, followed by frenectomy (24.4%) and frenuloplasty (17.3%); 26.7% reported no intervention (*p* < 0.001). Italy showed the highest rate of frenotomy (55.5%), whereas Brazil favored frenectomy (43.4%) and frenuloplasty (30.3%). Portugal reported the highest rate of non-performance (68.7%). Pediatric dentists most often performed frenectomy (35.5%) and frenotomy (32.3%), while oral surgeons preferred frenuloplasty (42.3%); general dentists and “Other” specialists most often reported no intervention. Scissors were the most common instrument used (37.2%), particularly in Brazil (72.0%), among pediatric dentists (42.1%), and oral surgeons (60.1%), whereas laser was most frequently employed in Italy (67.0%). Regarding anesthesia and pain control, combined topical and infiltrative anesthesia was most common (43.4%), although 26.4% reported using none—especially in Portugal (70.4%) and Chile (48.3%). Oral surgeons most often used infiltration with sedation (25.8%) and general anesthesia (16.6%) (Table 3).

**Table 3:** Knowledge and approach of ankyloglossia in Infants (>6 months).

Surgical Techniques	SPAIN	PORTUGAL	ITALY	BRAZIL	ECUADOR	CHILE
Frenotomy	28.40%	20.70%	55.50%	20.00%	40.00%	17.80%
Frenectomy	22.50%	07.30%	30.30%	43.40%	22.70%	20.10%
Frenuloplasty	20.30%	03.40%	14.20%	30.30%	21.40%	13.80%
Not perform	28.80%	68.60%	00.00%	06.30%	15.90%	48.30%
Total	100.00%	100.00%	100.00%	100.00%	100.00%	100.00%
Surgical Modalities						
Conventional	37.40%	12.80%	23.90%	72.00%	52.70%	24.10%
Laser	18.50%	14.50%	67.00%	12.00%	13.60%	18.40%
Electrosurgery	16.20%	05.10%	09.10%	10.30%	18.20%	09.80%
Not perform	27.90%	67.60%	00.00%	05.70%	15.50%	47.70%
Total	100.00%	100.00%	100.00%	100.00%	100.00%	100.00%
Pain control						
General Anesthesia	01.80%	00.60%	02.30%	00.00%	03.20%	15.50%
Immediate Breastfeeding	02.70%	00.00%	00.50%	00.60%	04.10%	00.60%
Local cold stimulant	01.40%	00.00%	01.40%	00.00%	00.90%	00.00%
Infiltrative local anesthesia	10.80%	06.10%	08.70%	09.70%	10.90%	04.60%
Infiltrative local anesthesia and sedation	11.20%	01.10%	13.80%	15.40%	08.20%	09.70%
Topical local anesthesia	06.80%	01.10%	16.40%	01.70%	02.70%	02.30%
Topical local anesthesia and infiltrative local anesthesia	38.30%	20.70%	54.60%	65.70%	57.70%	19.00%
Not use	27.00%	70.40%	02.30%	06.90%	12.30%	48.30%
Total	100.00%	100.00%	100.00%	100.00%	100.00%	100.00%

**Surgical Techniques****Surgical Modalities****Pain Control**X^2^ Tests


X^2^ Tests


X^2^ Tests



Valuedf*p*
Valuedf*p*
Valuedf*p*X^2^40915<0.001X^2^57515<0.001X^2^53735<0.001N1188

N1188

N1188



**Figure 1:**
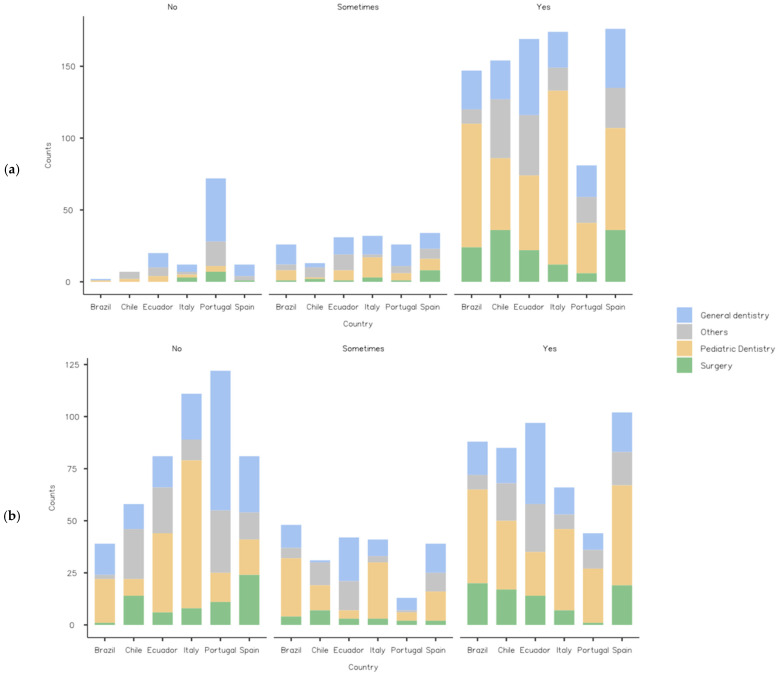
Recommended referral for adjunctive speech and myofunctional therapy in babies and infants. Shown here is an overview of the recommended referral pathway for adjunctive speech and myofunctional therapy in infants (**a**) and babies (**b**). The diagram highlights key indicators that may prompt referral, the roles of various clinicians involved in early evaluation, and the progression from screening to coordinated therapeutic intervention aimed at supporting optimal rehabilitation.

Recommendations for speech or myofunctional therapy were divided: 40.6% recommended it, 41.4% did not, and 18.0% recommended it conditionally. Pediatric dentists (45.1%) and oral surgeons (47.9%) recommended therapy more often than general dentists (33.8%) and “Other” professionals (35.7%). Collaboration in infants over six months was widely reported (75.8% regular), highest in Chile (88.5%) and Brazil (84.0%), and lowest in Portugal (45.3%), where 40.2% reported no collaboration. Pediatric dentists (88.3%) and oral surgeons (83.4%) were the most collaborative, while general dentists had the lowest collaboration (58.9%) and the highest non-collaboration (20.5%) (Figure 1).

## 4. Discussion

The diagnosis and management of ankyloglossia remain complex and inherently interdisciplinary, requiring collaboration among dental, medical, and allied health professionals. Despite ongoing efforts to standardize terminology and assessment, substantial variability persists in classification systems, diagnostic criteria, and treatment approaches, reflecting both significant research gaps and the absence of universal consensus across specialties [14,15]. In 2020, a panel of otorhinolaryngology experts proposed evidence-based definitions and management principles for ankyloglossia in children, underscoring the need for improved consistency in clinical practice [6]. Similarly, the AAPD recognizes that restrictive oral frenula can adversely affect breastfeeding, swallowing, and speech, highlighting the need for unified, evidence-based clinical protocols [11,16].

The present study assessed the knowledge and practices of 1188 dental professionals from six countries and revealed marked heterogeneity in diagnostic criteria, classification systems, and management of ankyloglossia. Although most professionals routinely examine the lingual frenulum (80.7%), fewer than half apply a standardized diagnostic tool (42.4%). This finding is consistent with previous studies showing that the absence of a universal classification system contributes to variability in prevalence estimates and treatment recommendations [3,6,7,9,10,17]. The reliance on functional assessments such as tongue mobility (19.5%) and the selective use of tools like BTAT (9.8%) or the Martinelli test (9.0%) reflects not only regional preferences but also differences in professional training and exposure to specific diagnostic methodologies.

Training emerged as a critical factor shaping diagnostic and therapeutic behavior. Although 67.2% of respondents reported specific training in ankyloglossia diagnosis, only half (50.6%) had received treatment training. This uneven educational background likely contributes to differences in clinical confidence and decision-making. Countries with higher training prevalence, such as Brazil and Spain, have historically incorporated frenulum assessment into pediatric or breastfeeding-focused continuing education programs, which may explain the higher consistency of clinical practices observed in these regions. In contrast, Portugal’s notably lower training rate suggests structural gaps in dental curricula or limited availability of specialized training opportunities. These educational discrepancies, also reported by other authors, further emphasize the need for harmonized, interdisciplinary training programs [7].

Another notable finding was the inconsistency in interdisciplinary collaboration. Although nearly half of participants reported collaborating with other health professionals, this practice was significantly more common among pediatric dentists and oral surgeons. Given that symptomatic ankyloglossia often presents as breastfeeding difficulties or early feeding dysfunction [18], limited collaboration with lactation consultants, pediatricians, and speech-language pathologists may delay appropriate referral and intervention. This finding aligns with prior research highlighting the benefits of structured multidisciplinary care in improving breastfeeding outcomes and functional rehabilitation [6,18].

Breastfeeding assessment showed considerable variation across countries despite its central role in diagnosing infant ankyloglossia. While 86.4% of clinicians assessed breastfeeding, the depth and consistency of evaluation differed, likely reflecting differences in training, scope of practice, and national breastfeeding support structures. Previous studies emphasize that early identification of symptomatic ankyloglossia—associated with poor latch, maternal nipple pain, and insufficient infant weight gain—is crucial for timely referral [2,18]. Thus, the variability found in this study underscores the need for standardized breastfeeding assessment protocols to support early and accurate referral.

With regard to management, early surgical intervention remained common, especially for infants under six months, with frenotomy being the predominant approach. These results align with evidence suggesting that timely frenotomy can alleviate breastfeeding-related symptoms [15,17]. The slightly higher surgical rate in older infants (>6 months) may reflect delayed diagnosis or an increasing reliance on frenectomy or frenuloplasty when functional restrictions persist. Instrument choice also displayed clear patterns: scissors were preferred over laser or electrosurgery. This preference likely reflects cost, accessibility, and the simplicity of the scissors’ technique rather than proven clinical superiority. Similar findings have been reported internationally [19].

Pain management practices showed marked inconsistency, reflecting gaps in standardized clinical guidance. The high proportion of clinicians performing infant frenotomy without anesthesia (32.3%) is consistent with literature indicating that the lingual frenulum is poorly innervated [20,21,22]. Yet, the variability across age groups and specialties—particularly the higher use of sedation by oral surgeons and the frequent absence of analgesia among general dentists—raises concerns regarding protocol inconsistency. Previous studies advocate for a multimodal approach to pain control tailored to age and procedure complexity, suggesting that clearer guidelines are needed.

Adjunctive therapies such as speech or myofunctional therapy were recommended by only 40.6% of professionals, despite strong evidence supporting their role in enhancing postoperative outcomes [23,24]. This underutilization suggests that frenotomy is still frequently perceived as a standalone intervention rather than part of a comprehensive functional rehabilitation plan. Prior research emphasizes that combining surgery with therapy significantly improves tongue mobility, breastfeeding effectiveness, and speech outcomes [19,23].

This study has several limitations. First, none of the participating countries share a standardized or regulated educational program on ankyloglossia, making cross-country comparisons of training and clinical preparedness challenging. Second, the sample included only dental professionals, excluding key members of the multidisciplinary team—such as pediatricians, speech therapists, midwives, and otolaryngologists—whose perspectives could provide a more complete understanding of clinical practice. Additionally, because participation was voluntary and the survey was distributed through professional networks, academic mailing lists, and social media, selection bias may have occurred. Dental professionals with greater interest, awareness, or clinical experience in ankyloglossia may have been more likely to participate, potentially limiting the representativeness of the sample.

Finally, the lack of international consensus on diagnostic and treatment criteria complicates comparisons, as each country follows its own professional guidelines and curricula.

In summary, this study underscores significant international variability in education, diagnosis, and management of ankyloglossia among dental professionals. Although awareness of the condition is widespread, the lack of standardized diagnostic tools, unified training pathways, and coordinated multidisciplinary care may contribute to variability in clinical decision-making. However, the present findings are based on self-reported professional practices and do not directly evaluate patient outcomes. By identifying specific gaps in training, diagnostic practices, and interdisciplinary collaboration across countries, these findings provide empirical evidence to guide the development of consensus guidelines, inform educational reforms, and support policy initiatives aimed at standardizing ankyloglossia care. Establishing international consensus guidelines and integrating evidence-based educational initiatives into dental curricula and continuing professional development would help improve diagnostic reliability, ensure appropriate treatment selection, and support comprehensive functional rehabilitation for affected infants.

## 5. Conclusions

This study highlights significant variability in the knowledge and clinical management of ankyloglossia among dental professionals across Spain, Italy, Portugal, Ecuador, Chile, and Brazil. These differences appear to be influenced by factors such as professional specialization, level and source of clinical training, and individual clinical decision-making processes regarding diagnosis and treatment in infants and young children.

Our findings indicate that examination of the lingual frenulum is not consistently integrated into routine dental check-ups. Furthermore, there is marked heterogeneity in diagnostic approaches, with a clear lack of consensus on assessment criteria and evaluation methods both within and between countries.

The absence of standardized clinical protocols and evidence-based guidelines contributes to inconsistent recognition and management of ankyloglossia. Such variability may result in both underdiagnosis and overtreatment, influenced by clinicians’ background, training, and interpretation of clinical signs.

Future research should prioritize the development and validation of standardized, evidence-based clinical guidelines for the diagnosis and management of ankyloglossia. Although our findings demonstrate substantial heterogeneity in current practice, further consensus-driven and outcome-based research is needed to determine which diagnostic and treatment approaches are most appropriate for standardization.

## Data Availability

The data presented in this study are available from the corresponding author upon reasonable request. The data are not publicly available due to privacy and ethical restrictions related to participant confidentiality.

## Abbreviations

The following abbreviations are used in this manuscript:

UIC	Universitat Internacional de Catalunya
AAPD	American Academy of Pediatric Dentistry
